# High-Precision Alignment Method for Electro-Optic Modulators via Combined Twyman-Green and Conoscopic Interferometry

**DOI:** 10.3390/s25196231

**Published:** 2025-10-08

**Authors:** Peng Zhang, Qi Lu

**Affiliations:** 1 Beijing Institute of Tracking and Telecommunication Technology, Beijing 100094, China; 2 Optical Testing and Characterization Center, High Power Laser Component Technology and Engineering Department, Shanghai Institute of Optics and Fine Mechanics, Chinese Academy of Sciences, Shanghai 201815, China; luqi@siom.ac.cn

**Keywords:** electro-optic modulators, alignment, Twyman-Green interferometry, conoscopic interferometry

## Abstract

**Highlights:**

**What are the main findings?**

- A combined Twyman-Green and conoscopic interferometry method is proposed to achieve high-precision alignment of electro-optic modulators (EOMs), simultaneously addressing optical axis, transmission axis, and crystal azimuthal alignment.
- The experimental results demonstrate alignment errors below 0.2862 mrad for alignment of polarizer/analyzer transmission axes and 0.3229 mrad for azimuth alignment of the electro-optic crystal, with sub-arcsecond repeatability validated via Bessel’s method.

**What is the implication of the main finding?**

- This method enables ultra-precise EOM alignment essential for emerging applications in quantum communication, lidar, and high-resolution imaging, where conventional techniques fall short.
- The proposed system offers a reproducible approach to alignment, potentially improving the performance and stability of advanced optical systems relying on EOMs.

**Abstract:**

Electro-optic modulators (EOMs) are critical components in advanced optical systems, including quantum communications and high-resolution imaging, where precise alignment is essential for optimal performance. However, conventional methods struggle to simultaneously achieve accurate optical axis, transmission axis, and azimuthal alignment of EOM components. This study proposes a high-precision alignment method that synergistically combines Twyman-Green and conoscopic interferometry. The Twyman-Green system first ensures precise optical axis alignment of the electro-optic crystal by minimizing tilt errors. Subsequently, under zero applied voltage, conoscopic interferometry is used to align the transmission axes of the polarizer and analyzer by verifying that the centroids of the interference features orient at 45° and 135°. Finally, under half-wave voltage, azimuthal alignment of the electro-optic crystal is achieved by ensuring the same centroid orientation. Experimental validation using a Z-cut LiNbO_3_ modulator demonstrates exceptional alignment accuracy, with root mean square errors below 0.2862 mrad for transmission axis alignment and 0.3229 mrad for azimuthal alignment. The proposed method offers a robust solution for high-precision EOM alignment in demanding applications.

## 1. Introduction

Efficient manipulation of the amplitude, phase and polarization light is the cornerstone of many modern optical applications. In the past decades, electro-optic modulators have served as practical technologies of choice to manipulate these properties of light and have been critical components in classical fiber-optic communication systems [1,2,3]. More recently, there has been increasing interest in electro-optic modulators due to their potential applications in emerging scientific areas such as quantum key distribution [4,5], free-space optical communications [6], wide-field fluorescence lifetime microscopy [7], and high-resolution three-dimensional imaging systems [8,9,10]. In classical optical communications, electro-optic modulators are generally utilized to manipulate the normal incident light beams. Nevertheless, in aforementioned emerging scientific areas, both normal incident and non-normal incident light beams must be manipulated by electro-optic modulators because the nature birefringence of electro-optic crystals is very sensitive to the propagation direction of the incident light, and minor alignment errors of electro-optic modulators can significantly impair the performance of these systems. It is of great importance to develop a method for electro-optic modulator alignment with high precision.

The alignment of optical components has been widely studied by researchers. Various methods have been proposed for high-precision optical axis alignment, including Fizeau interferometry [11], Twyman Green interferometers [12], fiber point diffraction interferometry [13], and pinhole point diffraction interferometry [14]. The inspection accuracy of these methods can reach a root mean square (RMS) of λ/60~λ/104 [15]. In addition, some technologies have been reported to characterize the misalignment errors in wave plates made of birefringent materials, including the rotating-compensator reflectance difference spectrometer [16], the rotating-compensator polarimeter [17], the three-spectrum method [18], and the Mueller matrix polarimeter [19]. However, existing methods struggle to simultaneously satisfy critical alignment requirements: the transmission axis alignment of polarizing components and the azimuth alignment of electro-optic crystals. In order to address these critical alignment challenges in emerging applications, a high-precision alignment method for electro-optic modulators that synergistically integrates Twyman-Green and conoscopic interferometry is proposed in this paper.

The remaining parts of this paper are organized as follows: Section 2 presents the alignment errors of a conventional electro-optic modulator, the alignment system and principle of the proposed method. The experimental results are reported in Section 3. Finally, the conclusions are presented in Section 4.

## 2. Methods

### 2.1. Alignment Errors

**Figure 1:**
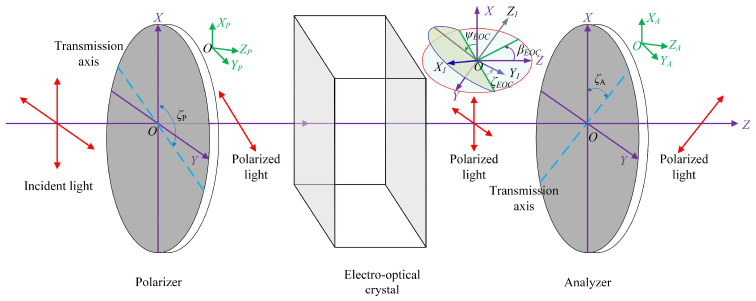
Schematic of alignment error of an electro-optic modulator.

The alignment errors of a conventional electro-optic modulator—comprising a polarizer, an electro-optic crystal, and an analyzer—are illustrated in Figure 1. The Cartesian coordinate system OXYZ is defined as the three-dimensional global coordinate system of the optical system, where the OZ-axis corresponds to the optical axis of the optical system, and OX and OY define the vertical and horizontal axes, respectively. For practical electro-optic materials such as Z-cut lithium niobate (LiNbO_3_), potassium dihydrogen phosphate (KDP), and potassium dideuterium phosphate (DKDP) crystals, the coordinate system OX_1_Y_1_Z_1_ defines the principal dielectric axes of the crystal. In this system, the OZ_1_ axis corresponds to the optical axis under zero applied voltage and is perpendicular to the OX_1_Y_1_ plane. Under ideal alignment conditions, the optical axis (OZ_1_) of the electro-optic crystal (EOC) should be parallel to the optical axis (OZ) of the optical system, while the principal dielectric axes (OX_1_ and OY_1_) should be parallel to the global vertical (*OX*) and horizontal (*OY*) axes, respectively. Under non-ideal alignment conditions, the horizontal alignment angle (β_EOC_), the vertical alignment angle (ψ_EOC_), and the rotation error angle (ζ_EOC_) are employed to denote different misalignment types of the electro-optic crystal. The relationship between the coordinate system OX_1_Y_1_Z_1_ and the coordinate system OXYZ can be described by the following matrix [20]:

(1) $$\begin{array}{l} \left(\begin{array}{ccc} {\hat{X}}_{1} & {\hat{Y}}_{1} & {\hat{Z}}_{1} \end{array}\right)=\left(\begin{array}{ccc} \hat{X} & \hat{Y} & \hat{Z} \end{array}\right)\cdot \left[\begin{array}{ccc} 1 & 0 & 0\\ 0 & \cos\beta_{EOC} & -\sin\beta_{EOC}\\ 0 & \sin\beta_{EOC} & \cos\beta_{EOC} \end{array}\right]\cdot \left[\begin{array}{ccc} \cos\psi_{EOC} & 0 & \sin\psi_{EOC}\\ 0 & 1 & 0\\ -\sin\psi_{EOC} & 0 & \cos\psi_{EOC} \end{array}\right]\\ \cdot \left[\begin{array}{ccc} \cos\zeta_{EOC} & -\sin\zeta_{EOC} & 0\\ \sin\zeta_{EOC} & \cos\zeta_{EOC} & 0\\ 0 & 0 & 1 \end{array}\right] \end{array},$$

where $\hat{X},\hat{Y},\hat{Z}$ and ${\hat{X}}_{1},{\hat{Y}}_{1},{\hat{Z}}_{1}$ are the unit vectors along the *X*-, *Y*-, *Z*-axis, and the *X*_1_-, *Y*_1_-, *Z*_1_-axies, respectively. Under ideal alignment conditions, *β_EOC_* = *ψ_EOC_* = ζ*_EOC_* = 0°.

The Cartesian coordinate systems *OX_P_Y_P_Z_P_* and *OX_A_Y_A_Z_A_* correspond to the interface normal coordinate systems of the polarizer and analyzer, respectively. Here, *OZ_P_* and *OZ_A_* represent the interface normals of the polarizer and analyzer, while *OX_P_* and *OX_A_* are used to describe the transmission axes of these optical components. The axes *OY_P_* and *OY_A_* maintain orthogonality with respect to the planes *OX_P_Z_P_* and *OX_A_Z_A_*, respectively.

Similarly to the methodology used for describing alignment errors of the electro-optic crystal, the horizontal alignment angle (*β_P_*), the vertical alignment angle (*ψ_P_*), and the rotation error angle (ζ*_P_*) are employed to denote different misalignment types of the polarizer. Correspondingly, the horizontal alignment angle (*β_A_*), the vertical alignment angle (*ψ_A_*), and the rotation error angle (ζ*_A_*) are employed to denote different misalignment types of the analyzer. For subsequent analysis, we consider the relative orientation relationships among the electro-optic crystal, polarizer, and analyzer under typical electro-optic modulator operating conditions. Under ideal alignment configuration where the polarizer and analyzer are perfectly aligned, the angular parameters are defined as: *β_P_* = *β_A_* = 0°, *ψ_P_* = *ψ_A_* = 0°, ζ*_P_*= 0°, and ζ*_A_*= 90°.

Therefore, three essential alignment tasks should be addressed during the alignment of the electro-optic modulator:

(1) Optical axis alignment: The electro-optic crystal, polarizer, and analyzer are adjusted to ensure their surface normal directions (*OZ*_1_-axis, *OZ_P_*-axis, and *OZ_A_*-axis) coincide with the optical axis direction (*OZ*-axis) of the optical system. Specifically, the horizontal alignment angles are adjusted as *β_EOC_* = *β_P_* = *β_A_* = 0°, and the vertical alignment angles as *ψ_EOC_* = *ψ_P_* = *ψ_A_* = 0°. In practical applications, the polarizer and analyzer exhibit low sensitivity to the incident light direction, where the reflection co-point method suffices for alignment requirements. However, the electro-optic crystal demonstrates remarkable sensitivity to the incident light direction. Consequently, significant effort is required to achieve precise optical axis alignment, ensuring that the optical axis of the electro-optic crystal coincides with that of the optical system. Specifically, the horizontal alignment angle of the electro-optic crystal is *β_EOC_* = 0°, and its vertical alignment angle is *ψ_EOC_* = 0°.
(2) Transmission axis alignment: The transmission axes of the polarizer (OXP-axis) and analyzer (OXA-axis) are precisely aligned to be parallel with the OX-axis and OY-axis of the optical system, respectively. Specifically, the rotation alignment angle of the polarizer is ζP = 0°, and that of the analyzer is ζA = 90°.
(3) Azimuthal alignment of the electro-optic crystal: The principal dielectric axes *OX*_1_ and *OY*_1_ of the electro-optic crystal under zero-voltage conditions are precisely aligned with the *OX*-axis and *OY*-axis of the optical system, achieving a rotation alignment angle of ζ*_EOC_* = 0°.

### 2.2. Alignment Principle and System

#### 2.2.1. Alignment Principle

For a laser beam incident on an electro-optic crystal, let α_G_ be the angle between the *OZ*-axis and unit Poynting vector $\hat{S}in$. Let *ϕ*_G_ be the angle between the *XOZ* plane and the plane of incidence. The unit Poynting vector of the laser can be expressed as [20]:

(2) $$\hat{S}in=\left(\begin{array}{ccc} \hat{X} & \hat{Y} & \hat{Z} \end{array}\right)\left[\begin{array}{c} \sin\alpha_{G}\cos\phi_{G}\\ \sin\alpha_{G}\sin\phi_{G}\\ \cos\alpha_{G} \end{array}\right],$$

The three-dimensional polarization vector of the incident laser can be expressed as:

(3) $$E_{0}=\left(\begin{array}{ccc} \hat{X} & \hat{Y} & \hat{Z} \end{array}\right)\left[\begin{array}{c} E_{0X}e^{i\delta_{0X}}\\ E_{0Y}e^{i\delta_{0Y}}\\ E_{0Z}e^{i\delta_{0Z}} \end{array}\right]=E_{0}e^{i\delta_{0}}{\hat{E}}_{0},$$

where $E_{0}e^{i\delta_{0}}$ denotes the amplitude of the polarization vector, while ${\hat{E}}_{0}$ represents the unit direction vector of the polarization vector which satisfies ${\hat{E}}_{0}\cdot {\hat{E}}_{0}^{†}=1$.

Before the laser beam enters the electro-optic crystal, the basis vectors for the s- and p-polarization states can be expressed as follows [20]:

(4) $${\hat{E}}_{s}=\frac{{\hat{S}}_{in}\times \hat{Z}}{\left|{\hat{S}}_{in}\times \hat{Z}\right|},$$

(5) $${\hat{E}}_{p}=\frac{{\hat{S}}_{in}\times {\hat{E}}_{s}}{\left|{\hat{S}}_{in}\times {\hat{E}}_{s}\right|}.$$

Due to the birefringence effect in the electro-optic crystal, the incident light splits into two beams (Beam A and Beam B) upon passing through the crystal. The electric field vectors of these two beams at the detector plane of a camera can be expressed as:

(6) $$E_{total,A}=P_{N}\ldots P_{1}E_{0},$$

(7) $$E_{total,B}=P_{N}\ldots P_{1}E_{0},$$

where **P_k_** (k = 1, 2, …, N) represents the three-dimensional polarization ray-tracing matrix of the k-th polarization optical element. The specific mathematical expressions for these matrices can be found in Ref. [20].

The electric field vector of the light received by the detector in the camera can be mathematically expressed as:

(8) $$E_{sum}=E_{total,A}+E_{total,B}.$$

**Figure 2:**
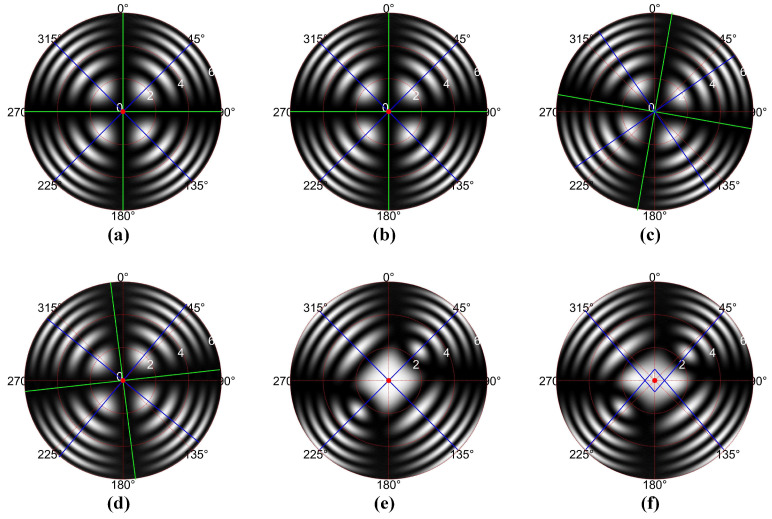
Conoscopic interference patterns under various applied voltages and alignment conditions. (**a**) The applied voltage is 0 V, and the electro-optic modulator has no alignment error ($\zeta_{P}=0^{{}^{\circ}}$, $\zeta_{A}=90^{{}^{\circ}}$, $\zeta_{EOC}=0^{{}^{\circ}}$); (**b**) The applied voltage is 0 V, and the electro-optic crystal has alignment error ($\zeta_{P}=0^{{}^{\circ}}$, $\zeta_{A}=90^{{}^{\circ}}$, $\zeta_{EOC}=10^{{}^{\circ}}$); (**c**) The applied voltage is 0 V, and the polarizer and analyzer have alignment errors ($\zeta_{P}=10^{{}^{\circ}}$, $\zeta_{A}=100^{{}^{\circ}}$, $\zeta_{EOC}=0^{{}^{\circ}}$); (**d**) The applied voltage is 0 V, and the analyzer has alignment errors ($\zeta_{P}=0^{{}^{\circ}}$, $\zeta_{A}=80^{{}^{\circ}}$, $\zeta_{EOC}=0^{{}^{\circ}}$); (**e**) The applied voltage is the half-wave voltage, and the electro-optic modulator has no alignment error ($\zeta_{P}=0^{{}^{\circ}}$, $\zeta_{A}=90^{{}^{\circ}}$, $\zeta_{EOC}=0^{{}^{\circ}}$); (**f**) The applied voltage is the half-wave voltage, and the electro-optic crystal has alignment error ($\zeta_{P}=0^{{}^{\circ}}$, $\zeta_{A}=90^{{}^{\circ}}$, $\zeta_{EOC}=10^{{}^{\circ}}$).

Based on the three-dimensional polarization vector and ray-tracing matrix, the conoscopic interference patterns under various applied voltages and alignment conditions can be obtained as shown in Figure 2, while the interface normals of the polarizer, analyzer, and electro-optic crystal are all aligned to be parallel to the optical axis of the measurement system. The conoscopic interference pattern shown in Figure 2a is obtained under the conditions that the transmission axis of the polarizer, the transmission axis of the analyzer, and the azimuthal orientation of electro-optic crystal are aligned with high precision ($\zeta_{P}=0^{{}^{\circ}}$, $\zeta_{A}=90^{{}^{\circ}}$, $\zeta_{EOC}=0^{{}^{\circ}}$), with the applied voltage on the electro-optic crystal being 0 V. This pattern comprises a set of concentric rings superimposed with a black Maltese cross. Two green lines marking the central axes of the Maltese cross in the conoscopic interference pattern are oriented along the horizontal (*OX*-axis, azimuthal direction of 0°) and vertical (*OY*-axis, azimuthal direction of 90°) directions. Compared with Figure 2a, Figure 2b shows the conoscopic interference pattern under the condition that the electro-optic crystal is misaligned ($\zeta_{P}=0^{{}^{\circ}}$, $\zeta_{A}=90^{{}^{\circ}}$, $\zeta_{EOC}=10^{{}^{\circ}}$). Nevertheless, the conoscopic interference patterns presented in Figure 2a,b exhibit identical configurations, indicating that the alignment error in the electro-optic crystal azimuthal orientation exerts no influence on the conoscopic interference pattern when the applied voltage on the electro-optic crystal is 0 V. In contrast with Figure 2a, the conoscopic interference pattern shown in Figure 2c is acquired under the condition that both the polarizer and the analyzer are misaligned ($\zeta_{P}=10^{{}^{\circ}}$, $\zeta_{A}=100^{{}^{\circ}}$, $\zeta_{EOC}=0^{{}^{\circ}}$). It is evident that the conoscopic interference pattern in Figure 2c exhibits a rotational shift relative to that in Figure 2a. The central axes of the Maltese cross are oriented along the azimuthal directions of 10° and 100°, respectively. Additionally, as shown in Figure 2d, the conoscopic interference pattern under the conditions of analyzer misalignment alone also exhibits rotational shift relative to that in Figure 2a. In conclusions, the conoscopic interference pattern demonstrates immunity to the azimuthal misalignment of the electro-optic crystal when the applied voltage on the crystal is 0 V, and the orientation of the central axes of the Maltese cross in the conoscopic interference patterns is determined by the transmission axes of the polarizer and analyzer. Meanwhile, as shown in Figure 2a, the centroids of the white area distribute on the blue lines oriented at 45° and 135°. This implies that the alignment of the transmission axes of the polarizer and analyzer can be verified by adjusting their orientation and analyzing whether the lines on which the centroids of the white area are distributed orient at 45° and 135°.

When the applied voltage on the electro-optic crystal is half-wave voltage, the crystal transits from uniaxial symmetry to biaxial symmetry. The conoscopic interference patterns under ideal alignment condition ($\zeta_{P}=0^{{}^{\circ}}$, $\zeta_{A}=90^{{}^{\circ}}$, $\zeta_{EOC}=0^{{}^{\circ}}$) and misalignment condition ($\zeta_{P}=0^{{}^{\circ}}$, $\zeta_{A}=90^{{}^{\circ}}$, $\zeta_{EOC}=10^{{}^{\circ}}$) are shown in Figure 2e and Figure 2f, respectively. Similarly to Figure 2a, the centroids of the white area in Figure 2e are distributed on the blue lines oriented at 45° and 135°. However, in contrast to Figure 2a, the central region of the conoscopic interference pattern in Figure 2e has transitioned from black to white, and the original concentric circular rings have transformed into elliptical structures. Compared with Figure 2d, under the conditions of azimuthal misalignment of the electro-optic crystal, the conoscopic interference pattern in Figure 2f exhibits distortion, and the centroids of the white area are no longer distributed on two lines oriented at 45° and 135°. Therefore, the azimuthal alignment of the electro-optic crystal can be achieved by capitalizing on the characteristic that under proper alignment conditions, the centroids of the white area in the conoscopic interference pattern form two distinct straight lines oriented at 45° and 135°.

#### 2.2.2. Alignment System

**Figure 3:**
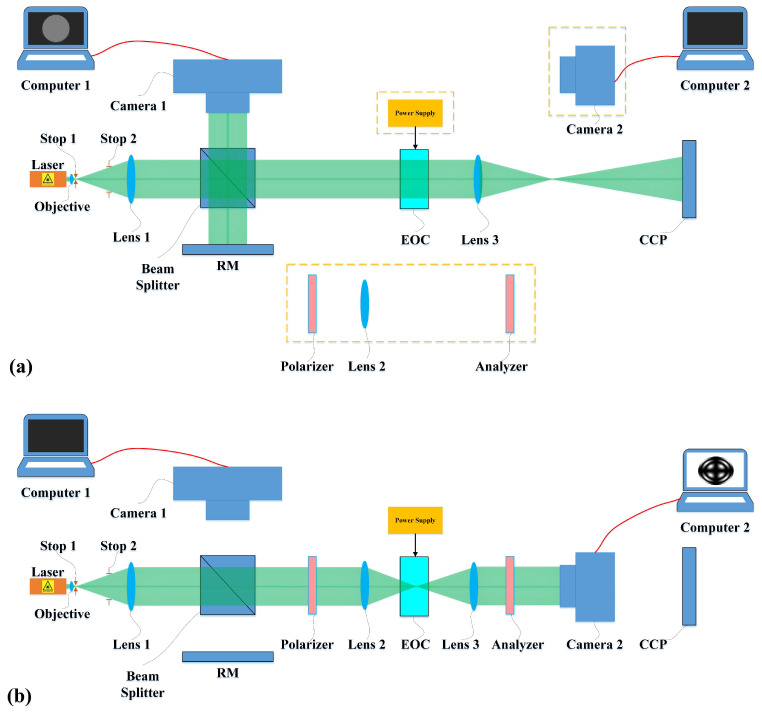
Optical layout schematics of the combined Twyman-Green and conoscopic interferometer. (**a**) Twyman-Green system and (**b**) Conoscopic interference system. RM, Reference Mirror; EOC, Electro-optic Crystal; CCP: Corner Cube Prism.

Section 2.2.1 introduces the fundamental principles for alignment of the transmission axes of the polarizer and analyzer, as well as the azimuthal alignment of the electro-optic crystal, based on conoscopic interference patterns, which is predicated on the prior alignment of the optical axes. While numerous methodologies exist for achieving optical axis alignment in optical components, an interferometric system integrating Twyman-Green and conoscopic interference techniques for precise alignment of the electro-optic modulator is proposed in this paper, with the optical configuration schematically illustrated in Figure 3.

The modified Twyman-Green interferometer configuration is shown in Figure 3a. In this setup, the laser beam first passes through an objective and through aperture stop 1, which serve to eliminate a portion of stray light while simultaneously expanding the laser beam. Subsequently, the divergent beam with a large divergence angle is collimated through lens 1, while the spot diameter is precisely controlled by aperture stop 2. The laser beam is split by the beam splitter prism into two beams that propagate in mutually perpendicular directions. One laser beam is directed onto the reference mirror (RM) and reflected, with the reflected light subsequently propagates through the beam splitter prism and is captured by the detector array of camera 1. During the alignment of the reference mirror, an autocollimator on the camera 1 side is used to ensure that the incident light is perpendicular to the reference mirror, with a perpendicularity error of less than 5 μrad. The other laser beam propagates along its original path, passing through the electro-optic crystal and lens 3 before being reflected by the corner cube prism. The reflected beam subsequently traverses lens 3 again, re-enters the electro-optic crystal, and finally reaches the detector array of camera 1 via the beam splitter prism. Finally, these two reflected laser beams interfere when arriving at the detector array of camera 1, and the resulting interference pattern is recorded by computer 1. The electro-optic crystal is precisely mounted on a high-accuracy three-dimensional rotation stage. During alignment procedures, adjusting the tilt angles of the crystal introduces varying optical path differences. This enables real-time observation of the interference patterns through computer monitoring. When null-interference fringes appear, the beam incident on the electro-optic crystal is perpendicular to the crystal’s surface, which is considered as the initial posture of the crystal, with an alignment error of less than 5 μrad.

Figure 3b illustrates the conoscopic interference system configuration. Compared with the setup shown in Figure 3a, this configuration requires the incorporation of four additional optical components into the optical path: the polarizer, lens 2, the analyzer, and camera 2. The polarizer and analyzer are both linear polarizers with a high extinction ratio, mounted in high-precision rotation mounts. Furthermore, the transmission axes of the polarizer and analyzer are mutually perpendicular to each other. The beam emerging from the analyzer is detected by camera 2, with the acquired images recorded by computer 2. Lens 2 serves to generate a convergent beam incident on the electro-optic crystal, while lens 3 is employed to convert the diverging beam emerging from the crystal into a collimated light. Additionally, the two lenses are confocal with each other.

Based on the preceding analysis, in order to achieve high-precision alignment of the electro-optic modulator, the alignment system can first be configured as a Twyman-Green interferometer to align the optical axis of the electro-optic crystal. Then, the alignment system is configured as a conoscopic interferometer, with the applied voltage on the electro-optic crystal being 0 V, and the directions of the polarizer and analyzer’s transmission axes are subsequently aligned. Finally, a half-wave voltage is applied on the electro-optic crystal to achieve precise azimuthal alignment of the crystal.

## 3. Experiments and Results

### 3.1. Experimental Alignment System

**Figure 4:**
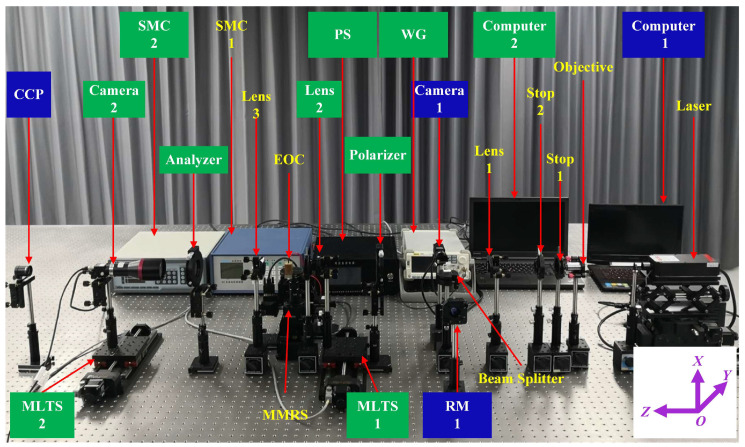
Experimental alignment system. RM: reference mirror; SMC: stepping motor controller; MMRS: multi-axis motorized rotation stage; WG: waveform generator; EOC: electro-optic crystal; PS: power supply; MLTS: motorized linear translation stage; CCP: corner cube prism.

The experimental alignment system designed in this study is illustrated in Figure 4, with distinct color-coding schemes applied to differentiate the optical components based on their measurement applications. The components exclusively employed for Twyman-Green interferometry are highlighted with blue backgrounds, while those specifically used in conoscopic interferometry are marked with green backgrounds. The components shared by both Twyman-Green interferometry and conoscopic interferometry are presented without background fill but distinguished by yellow text.

In Figure 4, the stepping motor controller 2 (SMC 2) drives both the motorized linear translation stage 1 (MLTS 1) and motorized linear translation stage 2 (MLTS 2), achieving a positioning accuracy of 2.5 μm. During the optical axis alignment of the electro-optic crystal using Twyman-Green interferometry, the experimental procedure requires the use of SMC 2 to coordinate the movement of MLTS 1 and MLTS 2 along the negative -axis direction, thereby removing Lens 2 and Camera 2 from the optical path. After completing the optical axis alignment of the electro-optic crystal, lens 2 and camera 2 are subsequently introduced into the optical configuration by moving MLTS 1 and MLTS 2 along the +Y-axis direction. The stepping motor controller 1 (SMC 1) is employed to actuate the multi-axis motorized rotation stage (MMRS) for precise adjustment of the electro-optic crystal, achieving a resolution of 1 arcsecond. The high-voltage DC power supply offers an adjustable output range of 0–5000 V, supporting both manual operation and external trigger modes. When utilizing the external trigger configuration, the system requires synchronization with an arbitrary waveform generator to initiate voltage application on the electro-optic crystal. This experiment is equipped with a CNILaser MSL-FN-671 laser (manufactured by CNI Laser, Changchun, China) and two Microvision cameras (manufactured by MicroVision, Beijing, China): an MV-EM040M (camera 1) and an MV-EM510M (camera 2). The charge-coupled device (CCD) detectors in camera 1 and camera 2 both operate in the frame exposure mode with 14-bit data output. The maximum resolutions are specified as 640 × 480 pixels for camera 1 and 2456 × 2058 pixels for camera 2. The laser employed in the experiment system is a single-longitudinal-mode laser. The output laser beam exhibits a wavelength of 671 nm with a beam diameter of approximately 1.5 mm. The apertures of lens 1, lens 2, and lens 3 are all 25.4 mm. Lens 1 (focal length: 200.7 mm) serves to collimate the divergent beam emerging from the objective and stop 1. Throughout the alignment procedure, a shearing interferometer should be systematically employed to verify and maintain strict collimation of the output beam. Lens 2 (focal length: 75.3 mm) serves to transform the collimated light into a convergent beam that is directed into the electro-optic crystal. Lens 3 shares an identical focal length with lens 2 and is configured in a confocal arrangement with the latter. The polarizer and analyzer are implemented as high-performance linear polarizers with extinction ratios exceeding 10^5^:1. To ensure precise alignment of the transmission axes for both the polarizer and analyzer, the linear polarizers are mounted on high-precision rotational stages. In our experimental setup, we employed micrometer-equipped rotational mounts for accurate transmission axis adjustment. A z-cut lithium niobite crystal with the size of 9 mm × 9 mm × 18.8 mm (X1 × Y1 × Z1) was chosen as the electro-optic crystal in the experiments.

### 3.2. Alignment Procedure

**Figure 5:**
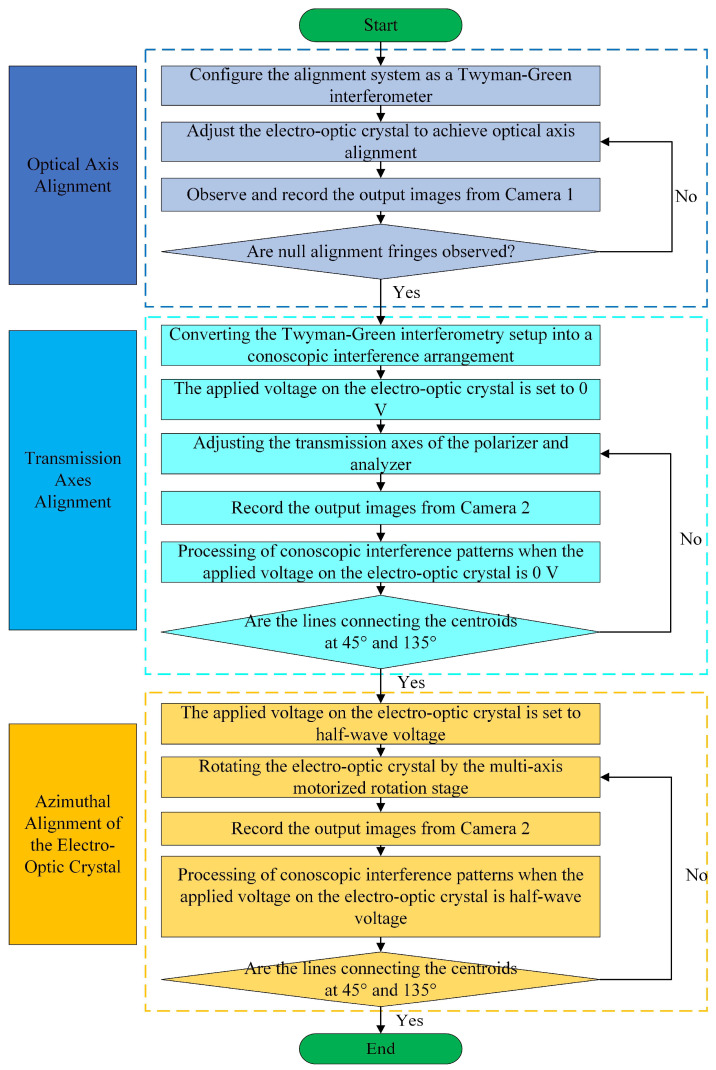
Workflow diagram of the alignment procedure for an electro-optic Modulator.

Figure 5 presents the schematic diagram of the electro-optic modulator alignment process. The experimental procedure involves three sequential steps: optical axis alignment, transmission axes alignment, azimuthal alignment of the electro-optic crystal.

#### 3.2.1. Optical Axis Alignment

The key experimental procedures for optical axis alignment are as follows:

(1) SMC 2 is employed to actuate MLTS 1 and MLTS 2, thereby removing lens 2 and camera 2 from the optical path along the negative Y-axis direction. This configuration ensures that the reflected light from both the reference mirror and the corner cube prism can be received by camera 1.
(2) The electro-optic crystal is mounted on a high-precision MMRS. First, the aperture of stop 2 is adjusted to its minimum size. The reflected spot of the laser is observed on the electro-optic crystal. Then, SMC 2 is used to control the MMRS for rotation around the X-axis, and slow adjustments are performed to make the reflected spot pass through the small aperture of stop 2 along the negative Z-axis direction, thereby achieving the preliminary alignment of the optical axis of the electro-optic crystal. Following this preliminary adjustment, fine-tuning is implemented through SMC 1 to drive the MMRS. Under MMRS control, the electro-optic crystal rotates around the X and Y axes. Throughout the alignment process, real-time monitoring of crystal orientation can be achieved by observing the optical images captured by camera 1.
(3) The output images from camera 1 are monitored on computer 1 to verify the presence of well-defined interference fringes. Optical axis alignment is achieved when the computer-monitored interference fringes progressively widen until they completely disappear.

#### 3.2.2. Transmission Axes Alignment

The key experimental procedures for transmission axes alignment are as follows:

(1) SMC 2 is activated to coordinate the motion of MLTS 1 and MLTS 2 along the +Y-axis direction. This mechanical adjustment positions lens 2 and camera 2 in the optical path, effectively transitioning the measurement configuration from Twyman-Green interferometry to conoscopic interferometry.
(2) The analyzer is positioned in front of the polarizer, with a high-precision laser power meter detecting the emergent beam from the analyzer. By rotating the analyzer around the Z axis, we minimize the detected optical power through angular adjustment. This alignment procedure establishes the perpendicular relationship between the transmission axes of the polarizer and analyzer, as confirmed by the power meter reaching its minimum reading.
(3) The adjusted analyzer is repositioned behind the electro-optic crystal. The transmission axes of the polarizer and analyzer are synchronously rotated around the Z axis with identical angular increments. With no voltage applied to the crystal, the conoscopic interference pattern generated by camera 2 is recorded using computer 2.
(4) The conoscopic interference patterns under zero-voltage conditions should be processed to analyze whether the centroids of the white area distribute on the two lines oriented at 45° and 135°. When misalignment occurs, iterative adjustments should be made to the transmission axes of both the polarizer and analyzer.

#### 3.2.3. Azimuthal Alignment of the Electro-Optic Crystal

The key experimental procedures for azimuthal alignment of the electro-optic crystal are as follows:

(1) The applied voltage on the electro-optic crystal is set to half-wave voltage, and the crystal should be precisely rotated by the multi-axis motorized rotation stage around the Z axis.
(2) The conoscopic interference patterns generated by camera 2 are digitally recorded by computer 2.
(3) The conoscopic interference patterns under half-wave voltage conditions should be processed to analyze whether the centroids of the white area distribute on the two lines oriented at 45° and 135°. When misalignment occurs, iterative adjustments should be made to the azimuthal orientation of the electro-optic crystal.

### 3.3. Experimental Results

#### 3.3.1. Twyman-Green Interferometry Experimental Results

**Figure 6:**
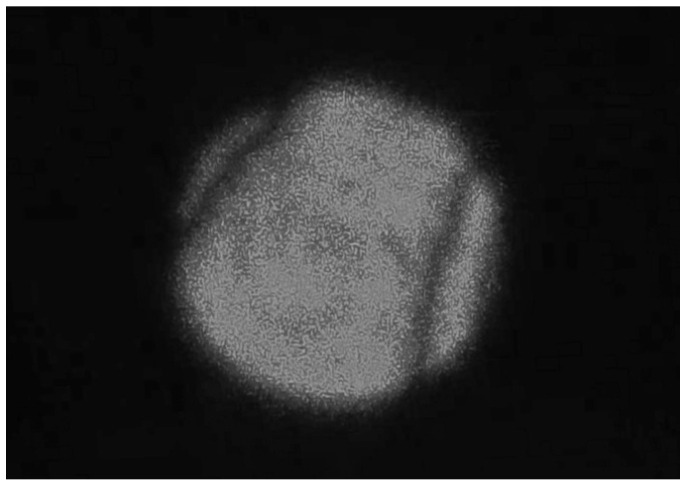
Interference pattern recorded during alignment of the electro-optic crystal’s optical axis using Twyman-Green interferometry.

Figure 6 presents the interference pattern recorded during the alignment of the electro-optic crystal’s optical axis using Twyman-Green interferometry.

#### 3.3.2. The Processed Conoscopic Interference Patterns

In this study, we developed a comprehensive algorithm for processing conoscopic interference patterns, aimed at automatically extracting the centroids of bright spots and analyzing their spatial distribution characteristics. The algorithm, implemented on the MATLAB 2017b platform, consists of three main modules: image preprocessing, feature extraction, and geometric analysis. The core processing workflow is elaborated in detail below.

The image preprocessing module serves as the foundational step of the algorithm. Initially, raw images are imported, followed by the application of Gaussian filtering for noise suppression. The standard deviation of the Gaussian filter was optimized and set to 2. Binarization is performed using the classic Otsu adaptive thresholding algorithm, which automatically determines the optimal segmentation threshold by maximizing inter-class variance based on the bimodal characteristics of the image grayscale histogram. Compared to traditional fixed-threshold methods, the Otsu algorithm adapts to varying illumination conditions, significantly improving segmentation robustness. Experimental results demonstrate that this method exhibits excellent performance in segmenting bright spots in conoscopic interference patterns, accurately distinguishing bright spot regions from the background.

During the morphological processing stage, a combination of opening and closing operations is employed. First, opening operations with a disk-shaped structural element effectively eliminate discrete noise points and smooth bright spot boundaries. This is followed by closing operations to fill internal holes in bright spots and connect fine fractures. The size of the structural element is selected based on statistical analysis of typical bright spot dimensions, ensuring the removal of noise while preserving the integrity of valid features.

In the feature extraction module, the algorithm begins by identifying all potential bright spot regions through connected component analysis, followed by filtering based on area thresholds to exclude noise interference. The geometric centroids of each bright spot region are then calculated, providing precise coordinate data for subsequent analysis.

The geometric analysis module introduces innovation through automated centroid grouping and line fitting. The algorithm first computes the average position of all centroid points as a reference center, then converts the Cartesian coordinate system to a polar coordinate system. Based on angular distribution characteristics, centroids are automatically grouped into two directional sets at 45° and 135°. The angular tolerance is set to 10°, a parameter systematically optimized to ensure grouping accuracy while accommodating varying degrees of image distortion. After sorting each group of centroid points by radial distance, least squares fitting is applied to achieve mathematically optimal line fitting.

The final parameter calculation includes the angle between the two fitted lines. The angle is computed using analytical geometry methods, accurately reflecting the symmetric characteristics of the conoscopic interference patterns. The algorithm also incorporates a robust exception-handling mechanism, providing appropriate error prompts when insufficient point sets or parallel lines are detected, thereby ensuring program stability.

**Figure 7:**
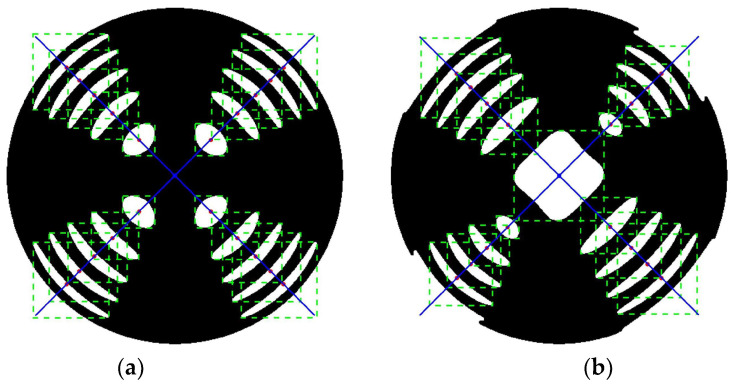
The processed conoscopic interference patterns: (**a**) the applied voltage on the electro-optic crystal is 0 V; (**b**) the applied voltage on the electro-optic crystal is half-wave voltage.

Figure 7 illustrates the processing outcomes under voltage-free and half-wave voltage conditions, where the blue markers represent the extracted centroids. The orientation vectors derived from the coordinate analysis are illustrated as blue reference lines, providing quantitative alignment references for electro-optic modulator alignment.

#### 3.3.3. Verification of Electro-Optic Modulator Alignment

To verify the alignment of the electro-optic modulator, after completing the optical axis alignment, transmission axis alignment, and principal dielectric axis alignment, lens 2 is removed from the optical path along the negative Y-axis direction of the optical system via MLTS 1, ensuring that the beam entering the electro-optic crystal remained collimated. A laser power meter (Thorlabs PM100USB, Thorlabs, Newton, MA, USA) is then placed between lens 3 and the electro-optic crystal to measure the power of the light exiting the crystal. The aperture of stop 2 is adjusted to ensure that all light emitted from the electro-optic crystal is captured by the power meter. The parameters of the lithium niobate crystal used in the experiment are listed in Table 1.

Based on the aforementioned parameters, the half-wave voltage of the lithium niobate crystal can be calculated as follows:

(9) $$V_{\pi }=\frac{\lambda }{2n_{o}^{3}\gamma_{22}}\frac{d}{L}\approx 2011.4V,$$

**Figure 8:**
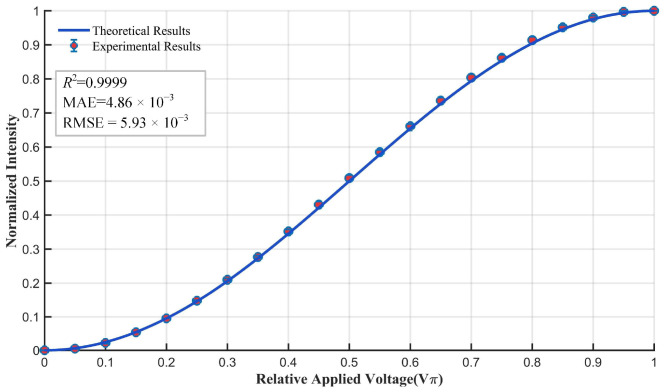
Comparison plot of the theoretical and experimental results of normalized intensity versus relative applied voltage. Theoretical normalized light intensity values (solid line) were calculated using the model described in Section 2.2 and reference [20], while experimental values (circular markers) were obtained by normalizing measured laser power under corresponding voltage conditions.

In addition, using the theoretical model described in Section 2.2 and reference [20], we calculated the normalized light intensity values under different relative applied voltage conditions. To facilitate comparative analysis, the experimentally measured laser power was normalized. The comparison between the experimental and theoretical results is shown in Figure 8. With a coefficient of determination (R^2^) = 0.9999, a mean absolute error (MAE) = 4.86 × 10^−3^, and a root mean square error (RMSE) = 5.93 × 10^−3^, the experimental results are in excellent agreement with the theoretical results, indicating the alignment method proposed in this paper achieves excellent alignment performance. Therefore, it can be concluded that the alignment of the optical axis, as well as the alignment of the transmission axis of the polarizer and the orientation axis of the electro-optic crystal, is sufficiently accurate. In subsequent analyses, the true values of the centroid alignment directions in the conoscopic interference pattern can be assumed to be 45° and 135°.

#### 3.3.4. Experimental Results for the Transmission Axes Alignment

To evaluate the alignment accuracy of the transmission axis alignment using conoscopic interferometry, twelve alignment experiments were systematically conducted on a lithium niobate electro-optic modulator. During the experimental procedure, stepper motor controllers were repeatedly employed to drive the two motorized linear translation stages (MLTS 1 and MLTS 2) for inserting and retracting lens 2 and camera 2 from the optical path. The manufacturer-specified repositioning accuracy of these translation stages was maintained below 0.1 μm throughout the experiments; thus, the measurement errors that could potentially arise from backlash effects during multiple positioning cycles are negligible.

**Figure 9:**
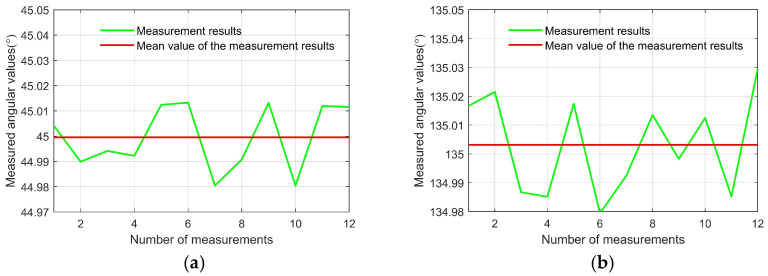
The alignment accuracy and measurement repeatability of the transmission axes alignment. (**a**) Measurement results of the first line. (**b**) Measurement results of the second line.

Figure 9 illustrates the orientation results of the two lines formed by the centroids of the white area in the conoscopic interference pattern when using conoscopic interferometry to align the transmission axes of the polarizer and analyzer. For the first line, the twelve repeated measurements showed a mean orientation angular of 44.9995° with a root mean square error of 0.0124° (0.2164 mrad).

The measurement repeatability evaluated using Bessel’s method is as follows:

(10) $$s\left(x\right)=\sqrt{\frac{\sum_{i=1}^{n}{\left(x_{i}-\bar{x}\right)}^{2}}{n-1}},$$

where $x_{i}$ denotes the result of the *i*-th measurement; *n* is the number of measurements; and $\bar{x}$ denotes the arithmetic mean of the measurement results, which can be expressed as:

(11) $$\bar{x}=\frac{1}{n}\sum_{i=1}^{n}x_{i}$$

Therefore, for the first line, the measurement repeatability evaluated using Bessel’s method yielded 0.0041° (0.0716 mrad). The second line demonstrated comparable performance, presenting a mean orientation angular of 135.0032°, with a root mean square error of 0.0164° (0.2862 mrad), across the twelve trials. Bessel’s analysis of measurement consistency for this orientation produced a repeatability index of 0.0055° (0.0960 mrad).

#### 3.3.5. Experimental Results for Azimuthal Alignment

**Figure 10:**
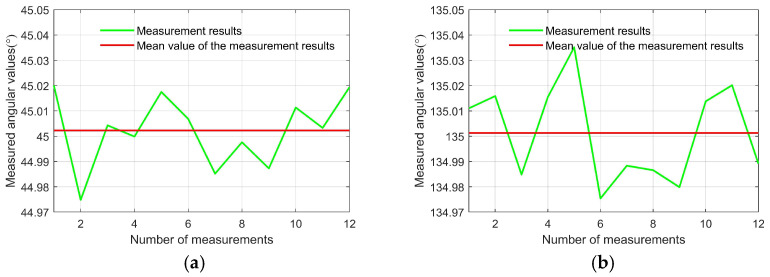
The alignment accuracy and measurement repeatability of the azimuthal alignment. (**a**) Measurement results of the first line. (**b**) Measurement results of the second line.

Figure 10 illustrates the orientation results of the two lines formed by the centroids of the white area in the conoscopic interference pattern when using conoscopic interferometry for azimuthal alignment of the electro-optic crystal. For the first line, the twelve repeated measurements showed a mean orientation angular of 45.0023° with a root mean square error of 0.0137° (0.2391 mrad). The measurement repeatability evaluated using Bessel’s method yielded 0.0046° (0.0803 mrad). The second line demonstrated comparable performance, presenting a mean orientation angular of 135.0013°, with a root mean square deviation of 0.0185° (0.3229 mrad), across the twelve trials. The measurement repeatability for this orientation was 0.0062° (0.1082 mrad).

## 4. Conclusions

A high-precision alignment method for electro-optic modulators which synergistically integrates Twyman-Green interferometry and conoscopic interferometry was proposed in this paper. Addressing critical alignment challenges in emerging quantum and imaging applications, the alignment system sequentially performs three tasks: optical axis alignment via Twyman-Green interferometry to minimize the tilt errors of the electro-optic crystal; alignment of the transmission axes of the polarizer and analyzer using zero-voltage conoscopic patterns to ensure the centroids of the interference features align at 45°/135°; and, finally, azimuthal alignment of the electro-optic crystal under half-wave voltage to ensure the centroids of the interference features align at 45°/135°. Experimental validation using a Z-cut LiNbO_3_ modulator demonstrated sub-arcsecond precision, with root mean square errors below 0.2862 mrad for the transmission axis alignment of the polarizer and analyzer and 0.3229 mrad for the azimuthal alignment of the electro-optic crystal. The measurement repeatability, evaluated via Bessel’s method, achieved values in the range of 0.0716–0.1082 mrad.

## Figures and Tables

**Table 1:** Parameters of the lithium niobate crystal.

Parameters	Values
Ordinary refractive index *n_o_* in the absence of an external electric field	2.2797
Length *L* in the direction of light propagation	18.8 mm
Thickness *d* in the direction of the external electric field	9 mm
Electro-optic coefficient ($\gamma_{22}$)	6.74 × 10^−12^ m/V

## Data Availability

The original contributions presented in the study are included in the article; further inquiries can be directed to the corresponding author.

## Abbreviations

The following abbreviations are used in this manuscript:

EOMs	Electro-optic modulators
RM	Reference Mirror
SMC	Stepping Motor Controller
MMRS	Multi-axis Motorized Rotation Stage
WG	Waveform Generator
EOC	Electro-optic Crystal
PS	Power Supply
MLTS	Motorized Linear Translation Stage
CCP	Corner Cube Prism
